# Systematics of *Trigonochloa* (Poaceae, Chloridoideae, Chlorideae)

**DOI:** 10.3897/phytokeys.13.3355

**Published:** 2012-06-29

**Authors:** Neil Snow, Paul M. Peterson

**Affiliations:** 1 527 S. Oakes, Helena, MT 59601, USA; 2 Department of Botany MRC-166, National Museum of Natural History, Smithsonian Institution, Washington, DC 20013-7012 USA

**Keywords:** Conservation, *Leptochloa*, leptotypification, Poaceae, systematics, taxonomy

## Abstract

A systematic treatment including descriptions and a key for identification is provided for the two species of *Trigonochloa*, a genus recently segregated from the polyphyletic *Leptochloa* s.l. *Trigonochloa* ranges from southern Africa east to India and Sri Lanka, reflecting the widely ranging *Trigonochloa uniflora*. *Trigonochloa rupestris* has a more limited distribution from East Africa to Yemen. *Trigonochloa* is diagnosable from other chloridoid grasses based on its unusually flaccid and membranous leaves that have uniquely enlarged lateral cells in the parenchyma sheath surrounding the vascular bundles in *Trigonochloa uniflora* (unconfirmed for *Trigonochloa rupestris* given limited material), primary and secondary vascular bundles that do not project above or below in fresh material, XyMS+ leaf anatomy, narrow spicate primary inflorescence branches, spikelets with one (or rarely two) florets, thinly membranous to hyaline lemmas, and a trigonous caryopses that bear a narrow but deep sulcus on the hilar side. Lectotypes are designated for *Agrostis montana* and *Cynodon gracilis*. The synonym *Leptochloa laurentii* De Wild. is confirmed for *Trigonochloa uniflora*.

## Introduction

The generic boundaries of *Leptochloa* P. Beauv. have been contentious more or less continuously since the genus was first described in 1812 (Valls 1978; McNeill 1979; Phillips 1982; Snow 1997). Many previous discussions of generic boundaries focused on whether *Diplachne* P. Beauv., often included in *Leptochloa*,could be recognized as a distinct genus (Phillips 1974; Jacobs 1987). A global monograph of *Leptochloa* (Snow 1997) based on morphology, lemmatal micromorphology (Snow 1996), anatomy of stems and leaves, and caryopsis morphology (Snow 1998) tested the monophyly of the genusin the context of a few putatively related genera. Preliminary results from cladistic studies using morphology were far from conclusive and at best suggested that *Diplachne* was not easily segregated from *Leptochloa* (Snow 1997). One noteworthy aspect of the cladistic studies (Snow 1997) was the consistent sister status of the *Leptochloa uniflora* Hochst. ex A. Rich. and *Leptochloa rupestris* C.E. Hubb. to other members of *Leptochloa* s.l., which was predictable since these two species share many morphological and anatomical characteristics absent in other members of *Leptochloa* s.l.

*Leptochloa* s.l. has been considered a diverse assemblage of C_4_ (nicotinamide adenine dinucleotide co-factor malic enzyme [NAD-ME] and phosphoenolpyruvate carboxykinase [PCK]) grasses in the tribe Chlorideae (Clayton and Renvoize 1986; Watson and Dallwitz 1992; Soreng et al. 2012) with approximately 32 annual or perennial species (Snow 1997, 2003). The range of morphological variation within many species of *Leptochloa* is significant (Snow 1997, Peterson et al. 2012). However, *Leptochloa* s.l. is demonstrably polyphyletic when tested with molecular DNA markers (Columbus et al. 2007; Peterson et al. 2010, 2012). In a large phylogenetic study of the Chloridoideae based on seven DNA sequence markers the species of *Leptochloa* s.l. were found to form three separate lineages (Peterson et al. 2010), and more recently using six DNA markers these species were found to form five separate lineages, each treated as a separate genus: *Dinebra* Jacq., *Diplachne*, *Disakisperma* Steud., *Leptochloa* sensu strictu, and *Trigonochloa* P.M. Peterson & N. Snow (Peterson et al. 2012). Peterson et al. (2012) found that *Trigonochloa uniflora* (Hochst. ex A. Rich.) P.M. Peterson & N. Snow and *Trigonochloa rupestris* (C.E. Hubb.) P.M. Peterson & N. Snow consistently resolved as a strongly supported clade (Peterson et al. 2012) outside of subtribe Elusininae.

*Leptochloa uniflora* was first described by Richard (1851).Chippindale (1955) later transferred this species to *Craspedorhachis* Benth., although her concept of *Craspedorhachis* was broader than that of other treatments (Clayton and Renvoize 1986; Gibbs-Russell et al. 1991; Watson and Dallwitz 1992). One current concept of *Craspedorhachis* includes three species that collectively occupy parts of sub-Saharan Africa and Madagascar (e.g., Simon et al. 2011).

Bentham (1882: 108) described two sections in *Leptochloa*, which he maintained distinct from *Diplachne*. In *Leptochloa* sect. *Pseudocynodon* Benth., characterized by spikelets having only one or two florets, Bentham ascribed *Leptochloa uniflora* and *Leptochloa neesii* (the latter of which included his concept of *Leptochloa polystachya* Benth.). The latter species, which is also characterized by one floret per spikelet (or in rare cases two), differs significantly from *Trigonochloa* by characters of leaf anatomy and has been transferred to *Dinebra* (Peterson et al. 2012).

The purpose of this paper is to present the systematics of *Trigonochloa* as the first step in revising *Leptochloa* s.l. into monophyletic genera.

## Materials and methods

Approximately 110 collections were analyzed from the following 15 herbaria: B, BM, BRI, CANB, K, M, MO, NY, P, PRE, S, TAES, US, W, Z. A few specimens previously determined incorrectly as *Leptochloa uniflora* (Gould and Soderstrom 1974; Snow 1997) have been identified subsequently as *Leptochloa neesii* Benth. [= *Dinebra neesii* (Benth.) P.M. Peterson & N. Snow] (Peterson et al. 2012). Geographical ranges are summarized using Brummitt (2001) and herbarium acronyms follow Thiers (2012).

Fresh leaf samples of *Trigonochloa uniflora* and *Leptochloa* s.l. were studied for, but not summarized, in Snow (1997). The first author also viewed black and white anatomical photographs of *Trigonochloa uniflora* taken by R. Ellis housed at PRE (Davidse & Ellis 5925, Ellis s.n., Ellis 3635, but see in particular Ellis 4534) and was able to confirm observations of *Trigonochloa uniflora* made previously by Metcalfe (1960). Stems (culms) were hand-sectioned with fresh or rehydrated material. The sources of anatomical vouchers are mostly at MO: *Trigonochloa rupestris*: Gilbert et al. 249, Pappi 2821; *Trigonochloa uniflora*: Snow & Burgoyne 6978, Siame 581, Ellis 2780, Davidse et al. 6643. Lemmatal micromorphology of *Trigonochloa* and other species of chloridoid grasses was studied using scanning electron microscopy (Snow 1996), and caryopses were studied using simple light microscopy (Snow 1998).

## Results and discussion

*Trigonochloa uniflora* and *Trigonochloa rupestris* are highly similar in gross morphology (Phillips 1974, 1995), lemmatal micromorphology (Snow 1996), stem and leaf anatomy, and caryopsis morphology (Snow 1998; Peterson et al. 2012). The genus differs from *Lopholepis* Decne., *Mosdenia* Stent, *Perotis* Aiton, and *Toliara* Judziewicz by having several to numerous unilateral, secund racemes scattered along a central axis rather than a single raceme or false spike (Watson and Dallwitz 1992; Judziewicz 2009). Species placed in *Lopholepis*, *Perotis*, and *Toliara* have 1-nerved lemmas whereas both species of *Trigonochloa* they are 3-nerved (Watson and Dallwitz 1992).

**Leaf anatomy.** The transverse anatomical features of the leaves of these two species differ in several significant ways from the rest of *Leptochloa* s.l. The leaf blades of both species are quite thin (and flaccid) when fresh in *Trigonochloa uniflora* and somewhat translucent. They also can be relatively broad basally and relatively short, thus appearing narrowly ovate.

Epidermal preparations of *Trigonochloa uniflora* made separately by the first author (*Davidse & Ellis 5925*, MO; unpublished) and by Roger Ellis (*Ellis 1928*; photos on herbarium specimen; PRE) show the adaxial (more so) and abaxial (less so) surfaces (apart from areas above bulliform cells) to be covered with narrow rows of relatively small cells, virtually all of which are capped by a centrally located and prominent papilla (Snow, unpubl.). In addition, the cells of the leaf blade epidermis and lemma surface are not always clearly differentiated into short and long cells (Snow 1996).

Keels (areas of parenchyma in the middle of the leaf blade lacking vascular bundles) are absent, or if present then small, and if present then lacunae within the parenchyma are absent. Primary and secondary vascular bundles differed only slightly in size and in fresh material do not project adaxially or abaxially. Bulliform cells were noted between adjacent vascular bundles. Colorless cells were not observed between vascular bundles, but they do occur adaxially to the primary and secondary bundles, and may be the only cell layer between the epidermis and the secondary vascular bundles. As many as five successive colorless cells (in cross section) were observed adaxially to a secondary vascular bundle on *Ellis 4534*. Metcalfe (1960: 285) reported only a single bundle sheath in *Leptochloa uniflora* and first noted the significantly enlarged parenchyma sheath cells at 3 and 9 o’clock, which he termed *lateral cells*, which we confirmed for this species (see in particular images with *Ellis 4534* at PRE). The lateral cells often penetrate deeply into laterally adjacent mesophyll and are nearly completely filled by a large chloroplast. Enlarged lateral cells were not clearly evident in the single specimen of *Trigonochloa rupestris* examined from limited rehyrdated material so we cannot yet confidently confirm nor reject their presence in this species.

Hattersley and Watson (1976: 303) reported the species as being XyMS-, based on the implied lack of intervening cells between the metaxylem and PCR sheath given the results of Metcalfe (1960). Valls (1978: 73–74) reported the presence of a double sheath for *Leptochloa uniflora* and reported that walls of the inner sheath were “exceptionally thin-walled”. Valls (1978), however, may have used specimens incorrectly identified that in actuality were *Leptochloa neesii*. Specimens studied by Roger Ellis, and seen for this study, confirm the observations of Metcalfe (1960) of the enlarged lateral cells and the presence of a double sheath, supporting the XyMS+ condition. However, the “outer sheath” cells only occur on the distal edges of the enlarged lateral cells, such that colorless cells typically occur adaxially and abaxially to the promixal part of the lateral cell (i.e., that part adjacent to the vascular bundle), with smaller chloroplast-bearing cells bearing smaller and more diffuse chloroplasts adjacent to the part of the lateral cell that occurs closest to the intervascular region. With permission while on site at PRE in 1996, the first author took 35 mm SLR photographs of the original black and white images of *Ellis 4534*, which show the leaf anatomy clearly. However, photos of the original images did not reproduce at a high enough quality to include in this paper. Other images of the leaf anatomy of *Trigonochloa* are unknown to the present authors.

**Stem anatomy**. Both species have a solid culm. *Trigonochloa uniflora* has inner and outer sclerenchymatous rings, although an inner ring was absent for *Trigonochloa rupestris*.

**Lemmatal micromorphology.** The two species share a unique combination of lemmatal micromorphological characters compared to other species of *Leptochloa* s.l. (Snow 1996). Cork cells and bicellular microhairs were present; macrohairs were terete (not crispate) and obtuse apically (not clavicorniculate; Snow 1996). Lemmatal characters in many species of *Leptochloa* s.l. but lacking in *Trigonochloa* included silica cells, and papillate long and papillate short cells (Snow 1996).

**Caryopsis morphology**. The caryopsis is trigonous in transverse section and possesses a narrow but deep hilar sulcus (=longitudinally grooved); the pericarp is tightly adnate (Snow 1998). This combination of caryopsis characters is unique among genera of chloridoids (Peterson et al. 2012). In our earlier molecular study *Trigonochloa uniflora* is sister to the *Mosdenia*-*Toliara*-*Lopholepis*-*Perotis* clade that lies outside of the subtribe Eleusininae (Peterson et al. 2012). Species of *Mosdenia* and *Perotis* have longitudinally-grooved caryopses that are dorsally or laterally compressed but not trigonous.

## Taxonomic treatment

### Trigonochloa

P.M. Peterson & N. Snow. Ann. Bot. 109: 1327. 2012.

http://species-id.net/wiki/Trigonochloa

#### Type species:

*Trigonochloa uniflora* (Chipp.) P.M. Peterson & N. Snow.

#### Description.

Plants annual to short-lived perennial, sometimes rhizomatous or stoloniferou. Culms (15–)35–130 cm long, terete in cross section, solid, decumbent or clambering to erect; nodes glabrous. Leaf sheaths half as long to slightly longer than internodes, glabrous or ciliate apically along margins; ligules 1–3.5 mm long, membranous, irregularly lacerate with age; leaf blades 1–13(–17) cm long, 0.3−14(–19) mm wide, linear to broadly ovate, flat, typically thin and flaccid, apex acuminate to acute. Panicles17−55 cm long, 2.0–8 cm wide, exserted at maturity, open, narrowly oblong to narrowly elliptic, composed of several to numerous unilateral, secund spikes or spicate racemes scattered along a central axis; rachis semi-terete; branches (1.5−)2–7 cm long, ascending, straight or slightly drooping. Spikelets 1.9−2.8 mm long, 1 (rarely 2-flowered), laterally compressed, subsessile, overlapping; disarticulation above the glumes; glumes 1.8−3.1 mm long, subequal, as long or longer than the floret, subequal, linear to narrowly ovate, 1-nerved, apex acute to acuminate, muronate or emucronate; lemmas 1.2−2.6 mm long, ovate, 3-nerved, thinly membranous to hyaline, minutely hairy along the nerves, apex acute, entire, awnless; paleas 1.5–2.5 mm long, keels ciliolate. Stamens 3. Caryopses 1−1.2 mm long, narrowly elliptic, trigonous in cross section, narrowly but deeply sulcate on the hilar side; surface smooth to slightly rugose-striate, light brown, pericarp fused, tightly adherent to endosperm. 2*n* = 36 for *Trigonochloa uniflora* (Gould and Soderstrom 1974).

#### Comments.

An appropriate common generic name to differentiate *Trigonochloa* from other members of *Leptochloa* is “triangle-seed grass”.

#### Key to species of *Trigonochloa*

**Table:** 

1	Leaf blades 0.3–4.0 (–5.0) mm wide, more or less linear to narrowly ovate; leaf sheaths margins minutely ciliate towards the apex, the collar never pilose	*Trigonochloa rupestris*
–	Leaf blades 5–14 (–19) mm wide, ovate to broadly ovate; leaf sheaths glabrous or sparsely pilose near collar but not ciliate along upper margins	*Trigonochloa uniflor*a

### Trigonochloa rupestris

(C.E. Hubb.) P.M. Peterson & N. Snow. Ann. Bot. 109: 1328. 2012.

http://species-id.net/wiki/Trigonochloa_rupestris

Figure 1A–E

Leptochloa rupestris C.E. Hubb., Bull. Misc. Inform. Kew: 195. 1941.

#### Type.

SOMALIA. Mt. Wobleh, J.B.Gillett 4981(holotype: K!; isotype: K!)

#### Description.

Plants perennial. Culms 35–85 cm tall, 0.7–1.4 mm wide at base, decumbent or sprawling-stoloniferous to erect, often arising from a thick root crown, branching (often profusely); internodes (0.7–)2.0–5.5(–8.5) cm long, soft to strongly lignified. Leaf sheaths longer or shorter than internodes, glabrous on sides but sometimes minutely papillate at high magnification, margins ciliate (trichomes <1 mm long); collar green; ligules 1.0–2.5 mm long, truncate or obtuse; blades 2.2–5.5(–10.7) cm long, 0.3–4.0(–5.0) mm wide, linear to narrowly ovate, glabrous above, glabrous below but sometimes densely minutely papillate, midrib prominent. Panicles 17–35 cm long, 2.0–4.5 cm wide with 13–66 branches, the branches (1.5–)2.0–4.5 cm long, minutely scabrous, the axils glabrous or at most scabrous and more or less short pilose on the exterior. Spikelets 2.0–2.8 mm long, 1-flowered, nearly sessile or with minute pedicels, more or less imbricate, callus area glabrous; lower glumes 2.4–3.1 mm long, membranous, lanceolate, midnerve scabrous, apex acute to acuminate; upper glumes, 2.2–2.8 mm long, otherwise like lower glumes; lemmas 1.2–2.1 mm long, ovate, light green or nearly white, the lateral nerves faint, sericeous along midnerve (use high magnification), the hair tips rounded, apex acute and awnless; paleas 1.6–2.0 mm long, hyaline, narrowly ovate, glabrous or sparsely sericeous near nerves, apex acute to obtuse; anthers 1.0–1.4 mm long, yellow to brownish green. Caryopses ca. 1.0 mm long and 0.4 mm wide, trigonous in cross section, the surface smooth.

**Figure 1.**
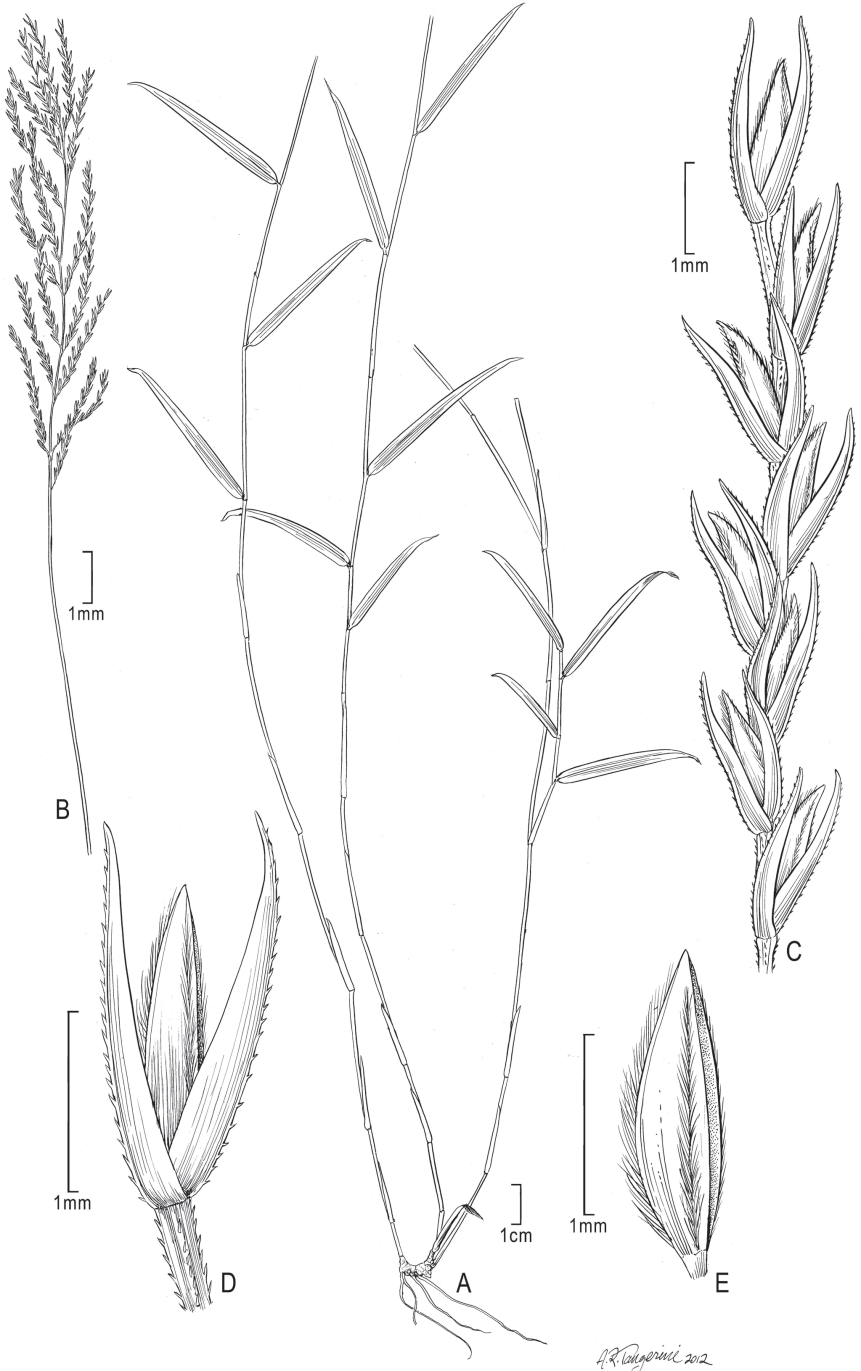
*Trigonochloa rupestris* (C.E. Hubb.) P.M. Peterson & N. Snow **A** Habit **B** Inflorescence **C** Portion of inflorescence branch **D** Spikelet **E** Floret. Drawn from *Wood 2000*.

#### Phenology.

Flowering June through January.

#### Distribution.

This species is found in Yemen and Eritrea south to Kenya in woodlands, hillsides, bushland and on damp rocks along streams; 900–1800 m. [Taxonomic Database Working Group (TDWG): 24: ERI, ETH, SOM; 25: KEN, UGA].

#### Conservation status.

Since many parts of its range are presently inaccessible to botanists or still remain inadequately surveyed this species is data deficient. Additional observations and collections are highly desirable.

#### Etymology.

The epithet *rupestis* is Latin for “of rocks”, presumably in reference to observations of the habitat of the type collection.

#### Vernacular name.

Somalian triangle-seed grass.

#### Comments.

This species closely resembles *Trigonochloa uniflora*, with which it is morphologically similar. The best character to recognize *Trigonochloa rupestris* from *Trigonochloa uniflora* is leaf blade width. However, its sprawling, branching, and perennial growth form with narrow culms typically distinguish it from *Trigonochloa uniflora*. The holotype and isotype are aberrant in their lack of ciliate sheath margins, but otherwise accord with the diagnostic characters. The observation of Phillips (1974) that *Trigonochloa rupestris* has more widely divergent leaf blades than *Trigonochloa uniflora* cannot be reliably applied to dried material. None of the specimens we have seen confirmed her observation (Phillips 1974) that *Trigonochloa rupestris* is rhizomatous (i.e., culms within the soil), but the stems can be somewhat sprawling and stoloniferous.

#### Specimens examined.

Eritrea. Donkollo, Schweinfurth 207 (P); Dongollo Presso Ghinda, Pappi 2821 (MO); Monte Dijot, Pappi 2940 (US). Ethiopia. Gamo Gofo: 44 km from Jinka on raod to Konso, ca. 3 km E of K’ey Afer, Gilbert et al. 8954(K); 13 km N of Lante, 29 km N of Arba Minch, Gilbert et al. 8874 (K). Arba Minch, Gilbert et al. 249 (K, MO). Harar:“Rock Valley”, 36 km along the road from Harrar to Jijiga, Amshoff 5520 (B, MO); Errer valley, 22 km SE of Harar on hwy to Djigdjigga, Burger 1162 (K); 7 km from Harar towards Jijiga, Gilbert & Gilbert 1443(K). Sidamo:Between the Genale Doria bridge and the main road Kebre Mengist-Neghelle, on the Biderre track, Friis et al. 1034 (K). Kenya. Rift Valley:West Suk Reserve, 30 mi N of Kitale, Bogdan 3429 (K); West Suk Reserve, 10 mi W of Kapenguria, Suam Riv. Valley, Bogdan 289(K); West Suk, Marech Pass, 40 mi N of Kapenguria, Bogdan 3844(K); 30 mi N of Nakuru, Bogdan 4891 (K, US); Kenya Grassland Research Station, Bogdan AB3964 (P). Somalia. “WOGR near Sheikh”, Wood S/72/95 (K); Jifa Uri, Gillett 4838 (K, US). Uganda.Northern: Moroto Mountains, Karamoja, Napper 1509(K); Warr, Karamoja, Thomas 3176(K). Yemen. Habash, Jebel Melhan, Wood 2848 (BM, K); 2 mi W of Mefhek, Wood Y/75/727 (BM); by Wadi Dur, Udayn, Wood 2000 (K, US).

### Trigonochloa uniflora

(Hochst. ex A. Rich.) P. M. Peterson & N. Snow. Ann. Bot. 109: 1328. 2012.

http://species-id.net/wiki/Trigonochloa_uniflora

Figure 2A–H

Leptochloa uniflora Hochst. ex A. Rich., Tent. Fl. Abyss. 2: 409. 1851.
Craspedorhachis uniflora (Hochst. ex A. Rich) Chippind., Grasses and Pastures of South Africa 205, f. 182. 1955.
Cynodon gracilis Nees ex Steud., Syn. Pl. Glumac. 1: 213. 1854. TYPE: India, Ab. loco. Wight Herbarium 8895 (lectotype: K! designated here, no specimen number given in the protologue; isotype: K!).
Agrostis montana Krock., Fl. Siles. 1: 110. 1787. *Agrostis montana* Rottl. ex Hook. f., Fl. Brit. Ind. 7: 298. 1896, nom. inval. TYPE: India, Tinnevelly; at Palamcotta, 28 Nov 1895, Rottler s.n. (lectotype: K! designated here).
Craspedorhachis menyharthii Hack. ex Schinz, Bull. Herb. Boissier, ser. 2, 1: 770. 1901. TYPE: Mozambique, Boruma, Tanuar, L. Menyharth 665 (lectotype: Z!, designated by Phillips 1974: 277 [who did not include the collection number]; duplicate of lectotype: W!).
Leptochloa laurentii De Wild., Miss. Em. Laurent i. 207. 1906. TYPE: Democratic Republic of the Congo, Kiri, E. Laurent s.n. (holotype: BR! [seen digitally, June 2012; barcode BR0000008761873]).

#### Type:

Ethiopia, In valle fluvi Tacaze, Schimper 1707(holotype: P!; isotypes: B!, BM!, GH!, K!, MO!, PRE!, S!, W!, photo ex W!)

#### Description.

Plants annual (or possibly weakly perennial). Culms (15–)45–130 cm tall, 0.6–2.0(3.0) mm wide at base, generally erect, sometimes geniculate below and stoloniferous by rooting at the nodes, sometimes branching, arising from fibrous roots or occasionally from a short knotted-rhizome; internodes 2–8 cm long, soft, solid. Leaf sheaths mostly shorter than internodes, glabrous throughout or rarely sparsely pilose near the collar, the margins glabrous; collars green; ligules 1.5–3.5 mm long, broadly obtuse, lacerate; blades 1–13(–17) cm long, 5–14(–19) mm wide, ovate to broadly ovate, glabrous above and below, midrib prominent or not. Panicles 25–55 cm long, 5–8 cm wide with 22–90 branches, the branches 2.5–7.0 cm long, minutely scabrous, the axils mostly glabrous internally but short pilose on external side. Spikelets 1.9–2.7 mm long, 1-flowered or rarely 2-flowered, but if so, only a few per plant, nearly sessile or with minute pedicels less than 0.3 mm long, somewhat imbricate, callus area glabrous; lower glumes 1.8–2.3 mm long, narrowly triangular, minutely scabrous on midnerve, apex acuminate to mucronate; upper glumes 2.2–2.6 mm long, otherwise like lower glumes; lemmas 1.6–2.6 mm long, ovate, whitish or light green, the lateral nerves very faint, sparsely pubescent along nerves, apex awnless; paleas 1.5–2.5 mm long, subequal to lemma, narrowly ovate, glabrous, apex obtuse or sometimes acute; anthers ca. 1 mm long, dark purple to pale olive green. Caryopses ca. 1.2 mm long and 0.4 mm wide, trigonous in cross section, the surface smooth to slightly rugose–striate. 2*n* = 36.

**Figure 2.**
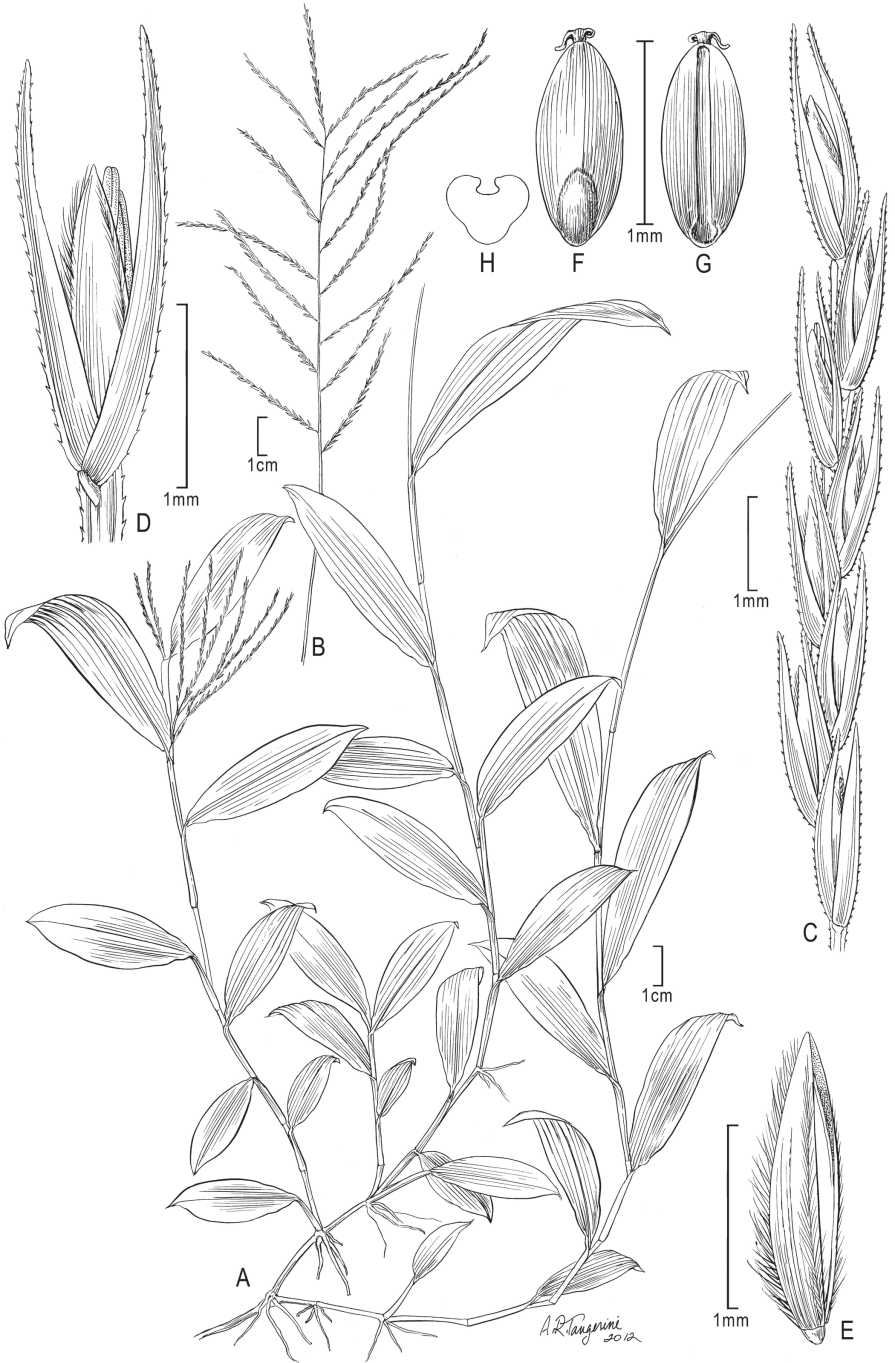
*Trigonochloa uniflora* (Hochst. ex A. Rich.) P.M. Peterson & N. Snow **A** Habit **B** Inflorescence **C** Portion of inflorescence branch **D** Spikelet **E** Floret **F** Caryopsis, dorsal view **G** Caryopsis, ventral view **H** Caryopsis, cross section. **A–C, F–G** drawn from *Ballard 1489*; **D, E** drawn from *Chare 4434*.

#### Phenology.

Flowering throughout the year when adequate moisture is available.

#### Distribtution.

This species isscattered through the eastern and southern portions of sub-Saharan Africa, rarely in India, most common in Sri Lanka in forests and shady areas on hillsides, well-drained and often sandy soils in disturbed and riparian areas; 0-1200 m. [TDWG: 22: GHA, NGR, ZAI (Dem. Rep. Congo); 25: KEN, TAN; 26: ANG, MLW, MOZ, ZIM; 27: BOT, NAM, NAT, TVL; 40: IND, SRL].

#### Conservation status.

Since the species is widespread it is of least concern (IUCN 2010) given its widespread occurrence. However, the typical size of populations is undocumented. The thinly membranous leaves likely are sought after by grazers, although the relative nutrition content of the leaves is unstudied.

#### Etymology.

The specific epithet is from the Latin *uniflora*, with reference to the single floret per spikelet.

#### Vernacular names.

Common triangle-seed grass. Kenya: Mkuse - Digo (Magogo and Glover 477, W).

#### Comments.

The species description and distribution differ from Snow (1997) because some specimens therein were incorrectly identified as *Leptochloa neesii*.

*Trigonochloa uniflora* individuals vary significantly in growth habit. Most specimens are relatively delicate, sprawling annuals, but some specimens have more erect, relatively stout culms that appear to be weakly perennial. The more erect forms typically occur in somewhat more open areas and have thicker leaves, whereas the more slender forms that frequently root at the lower nodes, typically occur in shade and have thinner leaves.

The glumes of *Trigonochloa uniflora* typically are longer than the single floret and may be mucronate. The caryopsis is sometimes dispersed with a tightly adnate lemma and palea, which may enhance water absorption prior to germination. The apex of the leaf sheath can sometimes be sparsely pilose, whereas in contrast nearly all specimens of *Trigonochloa rupestris* have ciliate sheath margins. Many specimens of *Trigonochloa uniflora* from Sri Lanka resemble *Trigonochloa rupestris* based on thin, sprawling culms. Two culms on Exell et al. 1060 (BM) have a sparse covering of papillose hairs on the upper and lower surfaces of the leaf blades.

Two counts of 2*n* = 40 ascribed to *Trigonochloa* (as *Leptochloa*) *uniflora* by Gould and Soderstrom (1974) were misidentifications of *Leptochloa neesii* (vouchers at US!). We confirm the voucher with apolyploid count of 2*n* = 36 based on *x* = 9 for *Trigonochloa (Leptochloa) uniflora* (Soderstrom and Kulatunge 1753, US!). In contrast, the count of *n* = 10 by Olorode (1975) has not been confirmed with a voucher.

#### Specimens examined.

Angola. Cuanza Norte: Cazengo, Welwitsch 2981 (BM, K); Cazengo, Gossweiler 4421 (BM, K); Granja de S. Luiz, Gossweiler 5200 (BM). Ab. loco, Gossweiler 2966(K) and 5444 (BM). Botswana. North-West: Riparian woodland, near Kasane,Blair Rains 67 (K, US).Dem. Repub. Congo.Haut-Katanga: Kibula, Callens 4776 (PRE). Kivu:Entre Nyangwe et Malela, Lebrun 5971 (PRE). Kongo Central: Kisantu, Vanderyst s.n. (US 889080). Tshuapa: Mpangu, Delhaye 440 (K). Province unknown: Gona, Vanderyst 5682 (US). Ghana. Ashanti: New Tafo, Lovi 3909 (K). Eastern:Aburi, Deighton 613 (BM). India. Kerala:“Palghat” [Palakkad], Madras Herbarium/South Indian Flora 16320 (US). Periakulam:Madurai, Matthew & Charles 51410 (K). Kenya. Coast: Longo Mwagandi Area, Shimba Hills, Magogo and Glover 477 (W); 50 mi SW of Mombasa, Shimba Hills, Bogdan AB3910 (P);Mombasa woodlands, Gibon s.n. (US 2954368); Kaya Muhaka, Luke 3405 (K); Forest between Umba and Mwena Rivers on Lungalunga-Msambweni Rd., Drummond & Hemsley 3787 (K, P). Malawi. Central:Dedza Distr., Mua-Livulezi Forest Reserve, Exell et al. 1060 (BM). Southern: Shire Valley, Hall-Martin 438 (MO); Lengwe Game Reserve, Hall-Martin 494 (K) and 582(K); Lengwe Game Reserve, NE corner, Brummitt 8884(K). Province Unknown: Mwenikumbila foothills, Jackson 1175 (MO). Mozambique. Manica:Maribane Forest, Chare 4434 (US); Amatongas Forest, near Gondola, Schweicherdt 272 (US). Sofala: Gorongosa N.P., Sangarassa Forest, 1 km W of Chitenga, Tinley 2497(K); Amatongas Forest, Schweicherdt 2341 (K, US). Zambezia: Malema, Torre & Paiva 11192 (PRE); Arredores de Mocuba, Torre 4908 (K). Namibia. Province unconfirmed. Mpilia Island, Killich & Leistner 3347 (K). Nigeria. Ondo: Idanre, Brenan & Jones 8731 (K). South Africa. Kwazulu-Natal:Mkuze Game Reserve, Ellis 3635 (PRE); Mkuze Game Reserve, parking lot by Bube (Kubube) Hide, Snow et al. 6978(MO, PRE); Tembe Elephant Park, Ward 1382 (PRE). Limpopo:Kruger N.P., Punda Milia area, Shipudza valley east of Punda Milia near Dongadziba, Ellis 3226 (K, PRE); Kruger N.P., ca. 12 km NW of Punda Milia, Davidse & Ellis 5925 (K, MO). Sri Lanka. Anuradhapura: Mihintale, Soderstrom & Kulatunge 1715 (CANB, K, TAES, US); Ritigala Strict Natural Reserve, ascent along eastern slope of Wannatikianda, Jayasuriya 1058(K, US). Central:54 miles N of Kandy toward Anuradhapura, trunk road A-9, marker 54/2, Gould 13250(US); Dambulla, Trimens 28(US); as previous, Ashton 998(K, US); Ruhuna N.P., Block I, Cooray 69030805R(US); Ruhuna N.P., Block I, Patanagala Camp, Clayton 5924 (CANB, K, TAES, US); Kumbukkan Oya, ca. 2 mi above mouth, at Megahakanda Meda Duwa Block 2, M–d Plot, R 16, Fosberg 51099 (US); Ruhuna N.P., Rugamtota on Menik Ganga (Plot 31), Fosberg & Mueller-Dombois 50192(US); Mennik Ganga (Riv.) 1 mile above Yalu Bungalow, Fosberg et al. 51045(US); Ruhuna N.P., Patanagala, Cooray 69120212R(K, US); Ruhuna N.P., Block 2, Cooray 69010502R(K, US); Ruhuna N.P., Block I, Rugamtota, Mueller-Dombois 69030704(US); Ruhuna N.P., Block I, next to Yala Camp site, Mueller-Dombois & Cooray 68013006(US); Ruhuna N.P., Block 1, in plot R13 between Andunoruwa and Komawa Wawa, Mueller-Dombois 69010713(US). Puttalam:Wilpattu N.P., Marai villu, Clayton 5597 (CANB, K, TAES, US). Trincomalee:Kantalai; road between Trincomalee and Kandy, Soderstrom & Kulantunge 1753 (CANB, K, TAES, US); Kantalai, 25 miles from Brincomalle on Kandy Rd., Ballard 1489(US). Tanzania. Iringa Region:Ab. loco, Greenway et al. 14075 (MO). Lindi Region:40 km W Lindi, Schlieben 5879 (B, BM, M, MO, S, US); Tendaguru, Migeod 104 (BM) and Migeod 126 (BM). Mahenge Region:Umgebung der Station Mahenge, Schlieben 1721 (BM, M, S). Morogoro Region:Uluguru-Gebirge, Schlieben 3630 (B, BM, G, M, S); 3 mi N Tunuguo, 30 mi SE Morogoro, Boaler 625 (B, US [2 sheets]). Province unknown.Rukwa Valley, Vuma Riv. near Zimba, Siame 581 (MO). Zambia. Central:Iolanda, N bank of R. Kafue, near Kafue town, Robinson 6440 (B, K, M); Kafue N.P., Musa-Kafue conflence, Mitchell 6/75(K). Eastern: Chikwa, ca 50 mi NW of Lundazi in Luangwa Valley, Robinson 822 (K, M). Northern:M’fume Camp, Verboom 922 (BM, K); Mporokoso,Lake Mweru-Wantipa,Richards 9117 (K, NY). Southern:Siburu forest, Sekute Chieftancy, Bainbridge 709 (BM); Victoria Falls, Crook 52602(K); Victoria Falls-Livingstone Island, Ellis 2780 (K, MO). Province unconfirmed. Kafue N.P., Mitchell 24/46 (B). Zimbabwe. Manicaland:Tanganda Tea Estate, Chipinga [now Chipinge], Brain 10615(K). Mashonaland West:Eastern Urungwe [=Hurungwe], tributary of upper Mauora, Phipps 868 (K). Urungwe,Chirundu, Simon 706 (BM). Masvingo: Bikita, 5 km E of Moodie Pass, Davidse et al. 6643 (BRI, K, MO, US). Matabeleleland North: Wankie [now Hwange], Kandahar Fishing Camp, Martin 87(K). Midlands: Gokwe, Sengua Research Station, Guy 2391(K).

## Supplementary Material

XML Treatment for Trigonochloa

XML Treatment for Trigonochloa rupestris

XML Treatment for Trigonochloa uniflora
